# Fellow-Workers and the Roll of Honour

**Published:** 1915-01-30

**Authors:** 


### ROUND THE WORLD.

[The Editor invites Early Information and Contributions Marked "Fellow-Workers."]

### Fellow-Workers and the Roll of Honour.

In cases of naval disasters one always hopes for some little time after the event that by some lucky chance or other yet a few more survivors may turn up. However, by now it can scarcely be doubted that the loss of the *Formidable* deprived the Naval Medical Service of three excellent officers, Messrs. G. Taylor, W. M. Mearns, and S. Hibbert.

The senior of these, Fleet-Surgeon Godfrey Taylor, was a Dublin man, who qualified M.B., Ch.B. in 1897. Soon after qualification he went to the City of Dublin Hospital as resident medical officer, and at the end of his experience there joined the Navy. Mr. Taylor had done well in his profession, but only enjoyed his rank of fleet-surgeon for little over a year before he lost his life on active service.

Surgeon William Mellis Mearns pursued his medical studies at Aberdeen, where he became an M.B., Ch.B. some six or seven years ago. Entering the Royal Naval Medical Service at the first opportunity, he was thus well within sight of promotion to the rank of staff surgeon when he joined his unfortunate ship last July.

His colleague on the *Formidable*, Surgeon Septimus Hibbert, was a Royal Naval Volunteer Reserve officer, having joined the R.N.V.R. about a year before the outbreak of war, and being appointed to the lost vessel when the fleet mobilised in July. An Oxford student, Mr. Hibbert did his clinical work at St. George's Hospital, and qualified M.B., B.Ch. Oxon. the year before last.

The London Hospital is represented on the staff of the King George Hospital by Dr. Lewis Smith and Mr. James Sherren; the former is a physician to out-patients at the "London," and was previously physician to the Poplar Hospital. Dr. Lewis Smith has had a distinguished career at the London Hospital since his early student days, when on taking his M.D. Lond. degree he qualified for the gold medal. He is the author of various papers and important articles on general medical subjects, as well as of a little book on "Golden Rules of Medical Practice."

Mr. James Sherren, F.R.C.S., is surgeon to the London Hospital and senior surgeon to the Poplar Hospital for Accidents. Some years ago he delivered the Erasmus Wilson lectures for the Royal College of Surgeons, where he at one time examined in anatomy for the "primary fellowship." In addition to important works in connection with abdominal surgery, Mr. Sherren is recognised as an authority on the surgical treatment of nerve injuries, a subject on which he has written a book. It is interesting to note that he is as at home on the sea as in the operating theatre, being, indeed, an experienced navigator.

Dr. Edward Farquhar Buzzard, another of the physicians appointed to the King George Hospital, is, of course, well known as a leading neurologist as well as a general consultant. A student of Oxford and St. Thomas's, he is now physician to out-patients at the latter institution, whilst he has for some years also been a member on the staff of the Hospital for the Paralysed and Epileptic in Queen Square. An M.D. Oxon., F.R.C.P. Lond., he has written largely on diseases of the nervous system, and has contributed comprehensive articles on special neurological problems to several of the foremost systems of medicine and treatment. Dr. Buzzard, who at one time was physician to the Royal Free Hospital and the Belgrave Hospital for Children, is also consulting neurologist to the Royal Free Hospital and the Hospital for Diseases of the Throat in Golden Square.

Dr. Raymond Henry Payne Crawfurd, who is a colleague of the above at the new hospital, is also an M.D. Oxon., F.R.C.P. Lond. A member of the staff of King's College Hospital, where he is physician and lecturer on clinical medicine, he was also at one time physician to the Royal Free Hospital and lecturer there on materia medica and pharmacology, a subject in which he recently examined for London University. Of late years Dr. Raymond Crawfurd has particularly delighted the fellow-members of his profession with interesting studies in the history of medicine, apropos of which he is the author of several notable lectures and works, the latter including "The King's Evil" and "Pestilence in Literature and Art."

Much regret has been felt at Guy's Hospital over the death of Lieut. Lionel Pengelly Waghorn, who was killed in action recently a few days after joining the Royal Berkshire Regiment, to which he had been transferred from the Royal West Kent Regiment. An old Marlborough boy, he went up to "Guy's" in 1909 and had passed his Intermediate M.B. Lond. and second conjoint examinations. On the outbreak of war he decided to interrupt his medical studies, although so near qualification, and volunteered for the Front. Having had experience as an officer of the Inns of Court Officers' Training Squadron he was given a commission, and laid down his life for his country at the early age of twenty-one years.

The vagaries of war time have brought about some remarkable contrasts of work. It is a far cry from practice in a pleasant suburb to the medical charge of a prison ship, an experience which has fallen to the lot of Dr. Athelstane Nobbs, the President of the Wandsworth Division of the British Medical Association. He is now medical officer on the *Scotian* at Portsmouth, with the rank of lieutenant in the R.A.M.C. Dr. Nobbs, who is an M.D., C.M. Edin., and a St. George's man also, has the medical care of over a thousand prisoners, including some of the sailors rescued from the German cruiser *Mainz*, which was sunk during the action off Heligoland.

The late Lieutenant Reginald Edward Porter left the London Hospital with the qualifications of M.B., B.S. Lond., L.R.C.P. Eng., in 1911, after having done well in the College. Immediately entering the Army Medical Service, he devoted himself keenly to his new work, and it is probable that, had he been spared, he would have eventually attained a high position as an Army surgeon.

